# Cytosolic mtDNA and associated EYA-mediated pro-inflammatory signaling modulate healthspan in Drosophila

**DOI:** 10.21203/rs.3.rs-7634140/v1

**Published:** 2025-10-13

**Authors:** David Walker, Ricardo Aparicio, Roberta Alessi, Agathe Solans, Matin Mojdeh, Vartika Sharma, Paul Oh, Matea Zelich, Toby Frank, Jae Hur

**Affiliations:** University of California, Los Angeles; University of California, Los Angeles; University of California, Los Anlgeles; University of California, Los Anlgeles; University of California, Los Anlgeles; University of California, Los Anlgeles; Harvey Mudd College; Harvey Mudd College; Harvey Mudd College; Harvey Mudd College

**Keywords:** cognitive function, Nuclease activity, DNautophagy

## Abstract

Mitochondrial dysfunction and pro-inflammatory signaling are each key drivers of aging. However, a clear understanding of the connections between mitochondrial homeostasis, inflammation and lifespan determination remains elusive. Upon mitochondrial stress or damage, mtDNA can be released into the cytosol thus encountering cytosolic DNA sensors and activating pro-inflammatory responses. Here, we report a striking age-related increase in cytosolic mtDNA, which can be counteracted by mitophagy, in *Drosophila* brain and muscle tissue. We find that upregulation of DNase II, an acid DNase which digests DNA in the autophagy–lysosome system, reduces cytosolic mtDNA levels in aged flies and prolongs healthspan. Reducing the abundance of cytosolic DNA in aged flies also dampens Rel/NF-κB pro-inflammatory signaling. Furthermore, we show that inhibition of EYA, a Rel/NF-κB-binding protein involved in immune sensing of DNA, in aging neurons counteracts brain aging and prolongs healthspan. Our findings identify DNase II and EYA as therapeutic targets to prolong healthspan.

## Introduction

The accumulation of dysfunctional mitochondria and persistent pro-inflammatory responses are each key hallmarks of aging^1^. Indeed, it is widely accepted that chronic inflammation, which has been called ‘inflammaging’^2^, contributes to the pathogenesis of age-related diseases limiting healthspan^3,4^. Potential mechanisms of inflammaging include changes to gut microbiota composition, intestinal barrier dysfunction, and chronic infections^5,6^. In addition, it is now apparent that sterile inflammation occurs in the absence of microorganisms and is typically associated with the recognition of intracellular debris released from damaged cells or organelles (also known as damage-associated molecular patterns; DAMPs)^7,8^. As mitochondrial dysfunction and inflammation are shared features of aging, it is interesting to speculate that mitochondrial-derived DAMPs may play a prominent role in inflammaging^9,10^. In support of this model, it is well-established that mitochondrial damage or dysfunction can lead to the release of mitochondrial DNA (mtDNA) which can activate innate immunity^11–17^. While the release of mtDNA from mitochondria is well established, the mechanisms allowing transfer to the cytosol are less clear^18^. There is an emerging understanding, however, that decreased mitochondrial membrane potential and increased mitochondrial permeability regulate the release of mtDNA into the cytosol^19^. On entering the cytoplasm, mtDNA can activate a plethora of different cytoplasmic DNA sensors and innate immune responses, including the cGAS/STING pathway, to trigger pro-inflammatory responses contributing to inflammatory pathology^17^.

Autophagy is a catabolic process in which cytoplasmic contents, including nucleic acids and organelles, are delivered to lysosomes for degradation^20^. A number of studies have reported that autophagy and/or mitochondrial autophagy (mitophagy) can restrain the innate immune response^9,21,22^. Mechanistically, there have been several reports that reveal an important role for autophagy/mitophagy in preventing the accumulation of cytosolic mtDNA-mediated inflammation^21–24^. A key concept that emerges from these studies is that mitophagy ensures the removal of damaged mitochondria and can, therefore, counteract the release of mtDNA into the cytosol and resulting inflammatory responses^22–24^. Furthermore, it has been shown that deletion of DNase II, which degrades mtDNA in the autophagy-lysosome system, predisposes to heart failure and cardiac inflammation in rodents^21^. Cytosolic mtDNA escaping from lysosomal degradation has also been shown to induce cytotoxicity in cultured cells and Parkinson’s disease phenotypes *in vivo*^25^. Recent studies have found that aging corresponds with the buildup of cytosolic mtDNA in certain cell types, such as rodent retinal cells, microglia, and *Drosophila* flight muscle^22,26^. Although the accumulation of cytosolic mtDNA has been linked to brain aging, retinal aging, and neurodegeneration^22,27^, a causal role for cytosolic mtDNA in organismal aging and lifespan determination is not well established. More specifically, experimental data showing that strategies to eliminate cytosolic mtDNA can slow organismal aging and/or prolong healthspan are lacking.

In this study, we have examined the role of cytosolic mtDNA and associated pro-inflammatory signaling in lifespan and healthspan determination. We show that there is an accumulation of cytosolic mtDNA in aging muscle and brain tissue of *Drosophila*. Inducing mitophagy, including in middle-aged flies, prevents the age-onset accumulation of cytosolic mtDNA. Critically, we show that upregulation of either DNase II or Stress Induced DNase (SID)^28–30^ can ameliorate cytosolic mtDNA accumulation during aging and prolong lifespan and healthspan. We also show that decreasing cytosolic DNA levels during aging, via DNase II or SID overexpression, dampens NF-κB-like proinflammatory signaling in aged flies. Recent work has shown that targeting the immune sensing of DNA, by inhibiting cGAS/STING signaling pathway, can reduce inflammation and improve tissue function in aged mice^27^. The Rel/NF-κB-binding protein EYA has also been shown to induce an innate immune response against cytosolic DNA in both flies and mammals^31,32^. However, the question of whether EYA contributes to age-onset inflammation and/or limits healthspan has not been addressed. We show that inhibiting EYA in aging neurons counteracts primary hallmarks of aging, as well as preventing synaptic aging and age-onset cognitive decline, leading to prolonged organismal healthspan. Our findings reveal that upregulation of nuclease activities, or inhibiting the immune sensing of DNA in neurons, during aging can prolong organismal health and longevity.

## Results

### Mitophagy counteracts cytosolic mtDNA accumulation in aged flies

**Figure 1:**
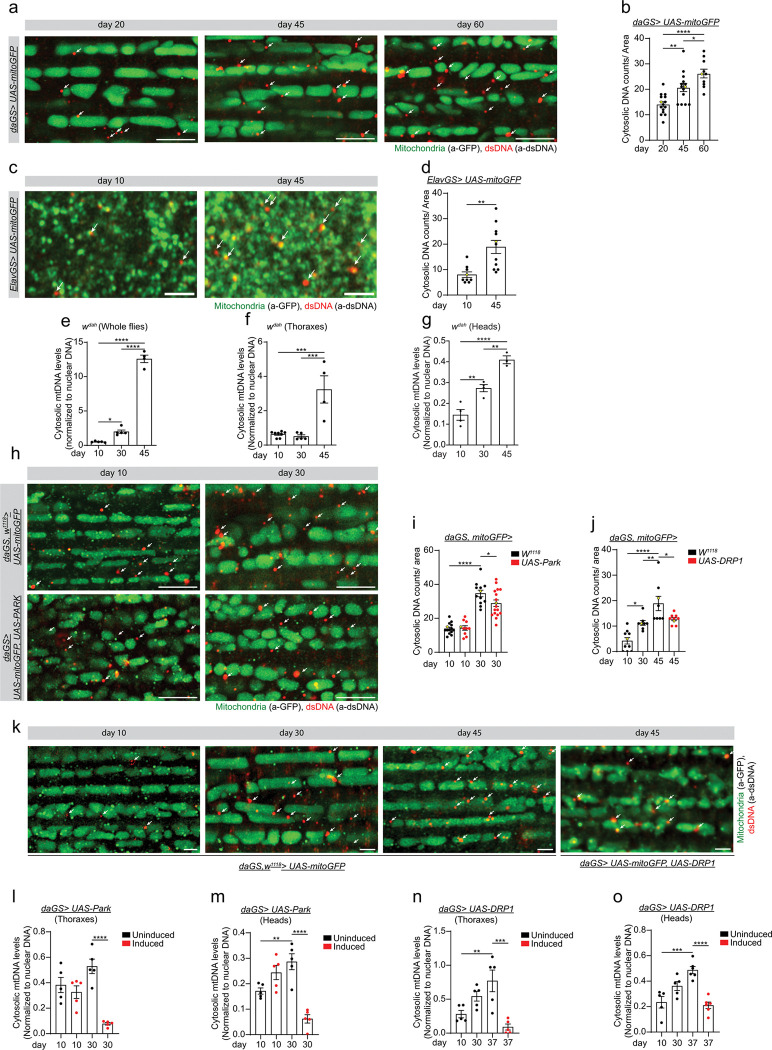
Mitophagy counteracts cytosolic mitochondrial DNA (mtDNA) accumulation in aged flies. **a)** Representative images (data point in yellow in b) of immunofluorescence staining of indirect flight muscles from young (day 20), middle-aged (day 45), and old (day 60) *daGS> UAS-mitoGFP* female flies with RU486-mediated transgene expression from day 3 onwards, showing dsDNA (red channel, anti-dsDNA) and mitochondria (green channel, anti-GFP). Scale bar 5mm. Arrows indicate dsDNA staining outside mitochondria. **b)** Statistical quantification of dsDNA foci outside of mitochondria as shown in a. d20 n = 15, d45 n = 14, and d60 n = 10 hemithoraxes. d20 vs d45 **p = 0.0039; d20 vs d60 ****p < 0.0001; d45 vs d60 *p = 0.0356. Ordinary one-way ANOVA test with Tukey multiple comparisons test. **c)** Representative images (data point in yellow in d) of immunofluorescence staining of *Drosophila* brains (mushroom body) from young (day 10), and old age (day 45) *elavGS> UAS-mitoGFP* female flies, showing dsDNA (red channel, anti-dsDNA) and mitochondria (green channel, anti-GFP). Scale bar 2 mm. Arrows indicate dsDNA staining outside mitochondria. **d)** Statistical quantification of dsDNA foci outside of mitochondria as shown in c. d10 n = 9, and d45 n = 11 brains. d10 vs d45 **p = 0.0021. Unpaired t-test. **e-g)** qPCR analysis of cytosolic mtDNA levels in wild type female flies in whole flies (e), thoraxes (f), and heads (g). **e)** d10 n = 5, d30 n = 5, and d45 n = 4 biological replicates with 20 flies per replicate. d10 vs d30 *p = 0.0119; d10 vs d45 ****p < 0.0001; d30 vs d45 ****p < 0.0001. Ordinary one-way ANOVA test with Tukey’s multiple comparisons test. **f)** d10 n = 8, d30 n = 5, and d45 n = 4 biological replicates with 20 thoraxes per replicate. d10 vs d45 ***p = 0.0002; d30 vs d45 ***p = 0.0002. Ordinary one-way ANOVA test with Tukey’s multiple comparisons test. **g)** d10 n = 4, d30 n = 4, and d45 n = 3 biological replicates with 25 heads per replicate. d10 vs d30 **p = 0.0058; d10 vs d45 ****p < 0.0001; d30 vs d45 **p = 0.0064. Ordinary one-way ANOVA test with Tukey’s multiple comparisons test. **h)** Representative images (data point in yellow in i) of immunofluorescence staining of indirect flight muscles from young (day 10), and middle-aged (day 30) *daGS> UAS-mitoGFP*, and *daGS> UAS-mitoGFP, UAS-Park* female flies with RU486-mediated transgene induction from day 3 to day 30, showing dsDNA (red channel, anti-dsDNA) and mitochondria (green channel, anti-GFP). Scale bar 5mm. Arrows indicate dsDNA staining outside mitochondria. **i)** Statistical quantification of dsDNA foci outside of the mitochondria as shown in h. *w*^*1118*^*, daGS> UAS-mitoGFP* d10 n = 14, and d30 n = 10, and *daGS> UAS-mitoGFP, UAS-Park* d10 n = 12, and d30 n = 17 hemithoraxes. d10 uninduced vs d30 uninduced ****p < 0.0001; d30 uninduced vs d30 induced *p = 0.0426. Ordinary one-way ANOVA test with Sidak’s multiple comparisons test. **j)** Statistical quantification of dsDNA foci outside of the mitochondria as shown in k. *w*^*1118*^*, daGS> UAS-mitoGFP* d10 n = 10, and d30 n = 8, and d45 n = 8, and *daGS> UAS-mitoGFP, UAS-DRP1* d45 n = 9 hemithoraxes. d10 vs d30 *p = 0.0222; d10 vs d45 ****p < 0.0001; d30 vs d45 **p = 0.0015; d45 vs d45 induced *p = 0.0453. Ordinary one-way ANOVA test with Sidak’s multiple comparisons test. **k)** Representative images (data point in yellow in j) of immunofluorescence staining of indirect flight muscles from young (day 10), middle-aged (day 30), and old (day 45) *w*^*1118*^*, daGS> UAS-mitoGFP* and *daGS> UAS-mitoGFP, UAS-DRP1* flies with RU486-mediated transgene induction from day 3 onwards for *daGS> UAS-mitoGFP* and from day 30 to 45 for *daGS> UAS-mitoGFP, UAS-DRP1*, showing dsDNA (red channel, anti-dsDNA) and mitochondria (green channel, anti-GFP). Scale bar 5mm. Arrows indicate dsDNA staining outside mitochondria. **l-m)** qPCR analysis of mtDNA levels in the cytosolic fraction compared to nuclei in *daGS> UAS-Park* female flies in thoraxes (l) and heads (m), with or without RU486-mediated transgene induction from day 3 to 30. **l)** d10 uninduced n = 5, d30 uninduced n = 5, d10 induced n = 5, and d30 induced n = 5 biological replicates with 20 thoraxes per replicate. d30 uninduced vs d30 induced ****p < 0.0001. Ordinary one-way ANOVA test with Sidak’s multiple comparisons test. **m)** d10 uninduced n = 5, d30 uninduced n = 5, d10 induced n = 5, and d30 induced n = 5 biological replicates with 25 heads per replicate. d10 uninduced vs d30 uninduced **p = 0.0091; d30 uninduced vs d30 induced ****p < 0.0001. Ordinary one-way ANOVA test with Sidak’s multiple comparisons test. **n-o)** qPCR analysis of mtDNA levels in the cytosolic fraction compared to nuclei in *daGS> UAS-DRP1* flies in thoraxes (n) and heads (o), with or without RU486-mediated transgene induction from day 30 to 45. **n)** d10 uninduced n = 5, d30 uninduced n = 5, d37 uninduced n = 5, and d37 induced n = 5 biological replicates with 20 thoraxes per replicate. d10 uninduced vs d37 uninduced **p = 0.0081; d37 uninduced vs d37 induced ***p = 0.0004. Ordinary one-way ANOVA test with Sidak’s multiple comparisons test. **o)** d10 uninduced n = 5, d30 uninduced n = 5, d10 uninduced n = 5, and d30 induced n = 5 biological replicates with 25 heads per replicate. d10 uninduced vs d37 uninduced ***p = 0.0002; d37 uninduced vs d37 induced ****p < 0.0001. Ordinary one-way ANOVA test with Sidak’s multiple comparisons test. RU486 or vehicle was provided in the media at a concentration of 25 mg/ml for panels a, b, c, d, j, k, j, n, and o; and 5 mg/ml for panels h, i, l, and m in the indicated treatment groups. Ethanol was used as control vehicle. Data are presented as scatter plots overlaying mean values +/− SEM.

Recent studies have reported an accumulation of cytosolic mtDNA in retinal cells and microglia of rodents^22,27^, and flight muscle of flies^26^. To validate and expand upon these findings, we used both immunofluorescence (IF) and qPCR-based approaches to examine cytosolic mtDNA levels in different tissues of aging *Drosophila*. We began by analyzing the accumulation of dsDNA in aging indirect flight muscles. Confocal analysis showed extramitochondrial dsDNA accumulation in aged flies compared to young flies (Supplementary Fig. 1a and quantification in b). To validate that the dsDNA antibody labels mtDNA we stained muscle of *daGS > UAS-mitoGFP* flies with antibodies against the mitochondrial transcription factor A (TFAM) and dsDNA. IF analysis shows that TFAM colocalizes with dsDNA antibody in young and old flies (Supplementary Fig. 1d). To expand our analysis, we adapted an IF staining approach using selective and specific permeabilization of cellular membranes to detect cytosolic dsDNA^33^. As this approach does not permeabilize mitochondria, it only allows detection of cytosolic DNA. Using this approach, we observed an age-related increase in dsDNA in muscle from young flies (day 20) to old flies (day 60) (Fig. 1a and quantification in b). Next, we sought to determine if cytosolic dsDNA accumulates in fly brain tissue. As in humans, *Drosophila* olfactory perception declines as a function of aging^34–36^. The mushroom body is a key structure for olfactory learning and memory, so we examined whether cytosolic dsDNA accumulates in the cytosol of mushroom body neurons. Confocal analysis showed dsDNA accumulation in aged mushroom body neurons compared to young flies (Fig. 1c and quantification in 1d). To confirm that cytosolic mtDNA accumulates during aging, we analyzed the levels of mtDNA-encoded genes in the cytosol of young (day 10), middle-aged (day 30), and old (day 45) wild type flies in whole flies, thoraxes, and heads by qPCR after cellular fractionation. First, we validated the fractionation approach using antibodies against mitochondrial proteins (Supplementary Fig. 1c). Using this fractionation approach, we found that the levels of the mtDNA-encoded genes COI (Citrate Oxidase I) and ND2 (mitochondrial NADH-ubiquinone oxidoreductase chain 2) were increased in the cytosolic fraction of old flies (day 45) versus young flies (day 10) in whole flies, heads and thoraxes (Fig. 1e–g, and Supplementary Fig. 1e and f). To determine whether the accumulation of cytosolic mtDNA was linked to an overall increase in mtDNA in aged flies, we analyzed the levels of total mtDNA in heads and thoraxes of wild-type flies. Interestingly, we observed a slight increase in total mtDNA levels in fly heads at day 30, followed by a decrease at day 45 (Supplementary Fig. 1h). We did not detect any variation in the levels of total mtDNA in thoraxes of wild type flies at any of the time points analyzed (Supplementary Fig. 1h). Together, these results demonstrate that mtDNA accumulates in the cytosol of *Drosophila* neurons and indirect flight muscles during aging.

We hypothesized that stimulating mitophagy may be an effective approach to counteract the accumulation of cytosolic mtDNA during aging. Indeed, recent work has reported that treating mice with Urolithin A, which can induce mitophagy, reduces cytosolic DNA in aged retinal cells^22^. Hence, we examined the ability of mitophagy induction to counteract cytosolic mtDNA accumulation in aged flies. The E3 ubiquitin ligase Parkin is known to play a key role in mitochondrial quality control and mitophagy^37^. Overexpression of Parkin can extend lifespan in flies^38^ and delay hallmarks of aging in several tissues and cell types in mammals^39^. In addition, we have shown that promoting dynamin-related protein 1 (Drp1)-mediated mitochondrial fission in midlife facilitates mitophagy and prolongs fly lifespan^40^. Hence, we analyzed the accumulation of cytosolic mtDNA during aging in *Parkin* and *Drp1* overexpressing flies and controls. We used the well-characterized *Drosophila* Gene-Switch system^41,42^ to overexpress Parkin and DRP1 in adult flies. This system allows both spatial and temporal control of the expression of the transgene of interest and the comparison of flies from the same cohort, since the only difference between control (uninduced) and experimental (induced) flies is the presence of the activator agent (RU486) or the diluent (ethanol). First, we examined, by IF, if ubiquitous overexpression of *Parkin* or *DRP1* could reduce the accumulation of dsDNA in flight muscle of old flies. As shown in wild type flies, control *daGS > UAS-Parkin* or *daGS > UAS-DRP1* flies accumulate dsDNA in the cytosol of aged muscle (Fig. 1h and k and quantification in i and j, respectively). Remarkably, 30 days of *Parkin* or 2 weeks of *Drp1* induction from midlife reduce the age-associated accumulation of dsDNA (Fig. 1h and k and quantification in i and j, respectively). To validate our findings, we analyzed the levels of cytosolic mtDNA in aged flies with and without *Parkin* or *DRP1* induction in heads and thoraxes by cellular fractionation and qPCR. mtDNA accumulates in the cytosolic fraction of *daGS > UAS-Parkin* and *daGS > UAS-DRP1* control flies in heads and thoraxes (Fig. 1l–o, and Supplementary Fig. 1i-l). Importantly, 30 days of *Parkin* or one-week of *Drp1* overexpression from midlife decreases the levels of the cytosolic mtDNA in heads and thoraxes of aged flies (Fig. 1l–o, and Supplementary Fig. 1i-l). RU486 treatment does not have any effect on cytosolic mtDNA levels during aging in control flies (Supplementary Fig. 1m). Together, these results show that mitophagy induction reduces the accumulation of cytosolic mtDNA in aged brain and flight muscles.

### Upregulation of nuclease gene activity during aging prolongs healthspan

**Figure 2:**
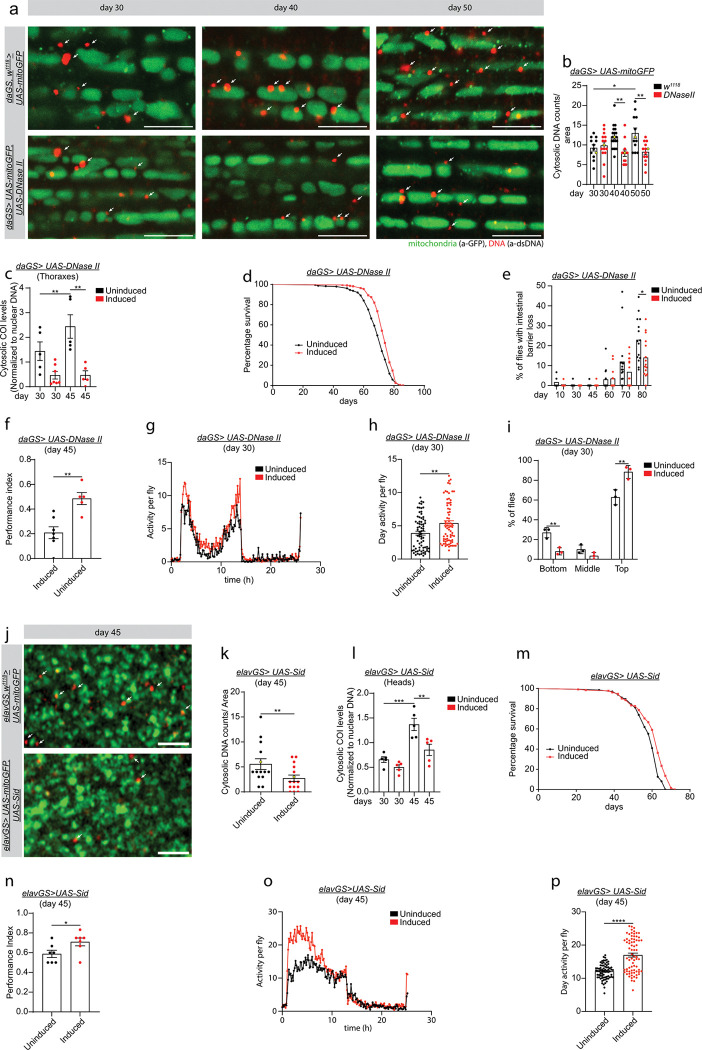
Upregulation of nuclease gene activity during aging prolongs healthspan. **a)** Representative images (data point in yellow in b) of immunofluorescence staining of indirect flight muscles from day 30, day 40, and day 50 *w*^*1118*^*, daGS> UAS-mitoGFP* and *daGS> UAS-mitoGFP, UAS-DNase II* female flies with RU486-mediated transgene expression from day 3 onwards, showing dsDNA (red channel, anti-dsDNA) and mitochondria (green channel, anti-GFP). Scale bar 5mm. **b)** Statistical quantification of dsDNA foci outside of the mitochondria as shown in a. Day 30 n = 12, day 40 n = 20, and day 50 n = 12 hemithoraxes in *w*^*1118*^*, daGS> UAS-mitoGFP*. Day 30 n = 15, day 40 n = 13, and day 50 n = 13 hemithoraxes in *daGS> UAS-mitoGFP, UAS-DNase II*. d30 uninduced vs d50 uninduced *p = 0.0311; d40 uninduced vs d40 induced **p = 0.0026; d50 uninduced vs d50 induced **p = 0.0022. Ordinary one-way ANOVA test with Sidak’s multiple comparisons test. **c)** qPCR analysis of mtDNA levels in the cytosolic fraction compared to nuclei in *daGS> UAS-DNase II* flies in thoraxes with or without RU486-mediated transgene induction from day 3 onwards. d30 uninduced n = 5, d30 induced n = 7, d45 uninduced n = 5, and d45 induced n = 5 biological replicates with 20 thoraxes per replicate. d30 uninduced vs d45 uninduced **p = 0.0011; d45 uninduced vs d45 induced **p = 0.0009. Ordinary one-way ANOVA test with Sidak’s multiple comparisons test. **d)** Survival curves of *daGS>UAS-DNase II* females with or without RU486-mediated transgene induction from day 3 onward. See Table S1 for statistical analysis. **e)** Percentage of flies with Intestinal barrier integrity loss during aging (Smurf assay) of *daGS> UAS-DNase II* females with or without RU486-mediated transgene induction since day 3 onward. n = 300 flies on day 10. d10 uninduced vs d80 uninduced ****p < 0.0001; d30 uninduced vs d70 uninduced ***p = 0.001; d30 uninduced vs d80 uninduced ****p < 0.0001; d45 uninduced vs d70 uninduced ***p < 0.0009; d45 uninduced vs d80 uninduced ****p < 0.0001; d60 uninduced vs d70 uninduced *p = 0.0342; d60 uninduced vs d80 uninduced * p = 0.0382; d60 uninduced vs d80 uninduced ****p < 0.0001; d70 uninduced vs d80 uninduced **p = 0.0011; d80 uninduced vs d80 induced *p = 0.0204. Ordinary one-way ANOVA test with Sidak’s multiple comparisons test. **f)** Performance index in olfactory aversion training at day 45 in *daGS>UAS-DNase II* female flies with or without RU486, assessed by the number of flies avoiding a shock-associated odor versus the total number of flies participating in the assay. Uninduced n = 7 and induced n = 5 vials with at least 15 flies per vial. **p = 0.0028. Unpaired t-test. **g)** Spontaneous physical activity of 30-day-old *daGS> UAS-DNase II* females with or without RU486-mediated transgene induction from day 3 to the day of the assay. **h)** Quantification of daytime physical activity of *daGS> UAS-DNase II* female flies with or without RU486 from day 3 to the day of the assay. Uninduced n = 3 and induced n = 3 vials with 10 flies per vial. **p = 0.0016. Unpaired t-test. **i)** Climbing index as a measure of endurance of 30-day-old *daGS> UAS-DNase II* female flies with or without RU486-mediated transgene induction from day 3 to day 30. Bottom represents the lower 1/3 of the cylinder, middle represents the middle 2/3 of the cylinder, and top represents the upper 1/3 of the cylinder. Uninduced n = 3 and induced n = 3 vials, with 3 vials containing at least 60 flies per vial. Bottom uninduced vs induced **p = 0.0041; top uninduced vs induced **p = 0.0013. 2-way ANOVA test with Sidak’s multiple comparisons test. **j)** Representative images (data point in yellow in k) of immunofluorescence staining of *Drosophila* brains (mushroom body) from day 45 *w*^*1118*^*, elavGS> UAS-mitoGFP* and *elavGS> UAS-mitoGFP, UAS-Sid* female flies with RU486-mediated transgene expression from day 3 onwards, showing dsDNA (red channel, anti-dsDNA) and mitochondria (green channel, anti-GFP). Scale bar 2mm. **k)** Statistical quantification of dsDNA foci outside of the mitochondria, as shown in a. Uninduced n = 14 and induced n = 15 brains. Uninduced vs induced **p = 0.0296. Unpaired t-test. **l)** qPCR analysis of mtDNA levels in the cytosolic fraction compared to nuclei in *elavGS> UAS-Sid* flies in heads with or without RU486-mediated transgene induction from day 3 onwards. d30 uninduced n = 5, d30 induced n = 5, d45 uninduced n = 5, and d45 induced n = 5 biological replicates with 25 heads per replicate. d30 uninduced vs d45 uninduced ***p = 0.0002; d45 uninduced vs d45 induced **p = 0.0036. Ordinary one-way ANOVA test with Sidak’s multiple comparisons test. **m)** Survival curves of *elavGS> UAS-Sid* females with or without RU486-mediated transgene induction from day 3 onward. See Table S2 for statistical analysis. **n)** Performance index in olfactory aversion training in 45-day-old *elavGS>UAS-Sid* female flies with or without RU486, assessed by the number of flies avoiding a shock-associated odor versus the total number of flies participating in the assay. Uninduced n = 7 and induced n = 7 vials with at least 15 flies per vial. Uninduced vs induced *p = 0.0442. Unpaired t-test. **o)** Spontaneous physical activity of 45-day-old *elavGS> UAS-Sid* females with or without RU486-mediated transgene induction from day 3 to the day of the assay. **q)** Quantification of daytime physical activity of *elavGS> UAS-Sid* female flies with or without RU486 from day 3 to the day of the assay. Uninduced n = 3 and Induced n = 3 vials with 10 flies per vial. Uninduced vs induced ****p < 0.0001. Unpaired t-test. RU486 or vehicle was provided in the media at a concentration of 25 mg/ml in the indicated treatment groups. Data are presented as scatter plots overlaying mean values +/− SEM.

To gain insight into the importance of cytosolic mtDNA accumulation during aging, we set out to determine whether interventions that reduce the levels of cytosolic DNA could be beneficial for organismal healthspan. First, we analyzed the expression levels of two enzymes with DNA degradation activity during aging: DNase II and Stress induced DNase (Sid). DNase II is a lysosomal enzyme that degrades DNA within the autolysosome^29,30^. Sid is an evolutionarily conserved enzyme that degrades both single and double-stranded DNA/RNA^28^. First, we analyzed the expression profile of *DNase II* and *Sid* in heads and thoraxes of wild type flies. *DNase II* mRNA levels do not change in heads or thoraxes of wild type flies during aging (Supplementary Fig. 2a and b). However, qPCR analysis shows that *Sid* transcript levels decrease in heads of wild type flies with age but does not vary in thoraxes (Supplementary Fig. 2h and i). To investigate the potential role of DNase II and Sid in degrading cytosolic mtDNA, we generated DNase II and Sid transgenic flies. We used the Gene-Switch system to overexpress *DNase II* and *Sid* with the ubiquitous driver *daughterless-GS* (*daGS*) and the neuronal specific driver *elavGS*, respectively. First, we validated the expression of the transgenes in thoraxes of *daGS > UAS-DNase II* and heads of *elavGS > UAS-Sid* flies. *DNase II* levels were upregulated by approximately 4-fold in thoraxes of young, middle, and old-age *daGS > UAS-DNase II* overexpressing flies (Supplementary Fig. 2c). *Sid* mRNA transcripts were upregulated by approximately 4-fold at day 30 and 6-fold at day 45 (Supplementary Fig. 2j). Next, we analyzed the accumulation of cytosolic dsDNA in *DNase II* and *Sid* overexpressing flies by using selective and specific cellular membrane permeabilization, without permeabilizing mitochondria. Upregulation of *DNase II* reduces the cytosolic dsDNA accumulation in aged muscles (Fig. 2a and quantification in b). In a complementary approach, we analyzed by cellular fractionation and qPCR analysis the levels of mtDNA-encoded genes in the cytosol of *DNase II* overexpressing flies. *DNase II* upregulation resulted in a reduction in mtDNA levels in the cytosol of thoraxes and heads on day 45 as compared to controls (Fig. 2c, and Supplementary Fig. 2d-f). Moreover, we found that neuronal *Sid* induction reduces the age-associated accumulation of cytosolic dsDNA at day 45 in *Drosophila* mushroom body neurons (Fig. 2j and quantification in k). Finally, we quantified the levels of cytosolic mtDNA genes by qPCR in *Sid* overexpressing flies. Figure 2 shows that cytosolic mtDNA accumulates in *elavGS > UAS-Sid* control flies and that neuronal *Sid* upregulation reduces by 50% the age-associated cytosolic mtDNA accumulation on day 45 as compared to control flies (Fig. 2l, and Supplementary Fig. 2k). These results demonstrate that induction of either of these DNA degrading enzymes reduces the age-associated accumulation of cytosolic mtDNA.

To characterize the effects of *DNase II* and *Sid* induction on *Drosophila* health, we analyzed the longevity of *daGS > UAS-DNase II* and *elavGS > UAS-Sid* female flies. Ubiquitous upregulation of *DNase II* or neuronal *Sid* induction each extends *Drosophila* median lifespan in several trials (Fig. 2d and m, and Table s1 and s2). Next, we examined whether nuclease-mediated lifespan extension is associated with improvements in healthspan. First, we determined whether *DNase II* induction improves intestinal barrier integrity during aging. Loss of intestinal barrier integrity is a well characterized evolutionarily conserved pathophysiological hallmark of aging associated with inflammation, frailty, and mortality^6^. In *Drosophila*, intestinal barrier dysfunction can be quantified by the “Smurf assay”^43,44^. Remarkably, we observed that *DNase II* induction delays the loss of intestinal barrier integrity in aged flies (Fig. 2e). To assess if *DNase II* and *Sid* induction could improve brain function in aged flies, we tested associative learning and memory using olfaction aversion training^45^. Briefly, *DNase II* and *Sid* overexpressing flies were exposed to a neutral odor (3-octanol, OCT) with a series of electric shocks. After one hour of rest, flies were placed in a T-maze and allowed to choose between OCT and a second neutral odor (4-methylcyclohexanol). Aged *DNase II* and *Sid* overexpressing flies perform better than their age-matched control flies in this assay (Fig. 2f and n). Next, we set out to determine if *DNase II* and *Sid* overexpressing flies showed improved locomotor activity and climbing ability. First, we analyzed spontaneous activity during 24 hours of *daGS > UAS-DNase II* and *elavGS > UAS-Sid* overexpressing flies. As shown in Fig. 2, ubiquitous *DNase II* and neuronal *Sid* induction increased daytime activity without affecting sleep compared to their respective age-matched controls (Fig. 2g, h, o, and p, respectively). Second, we analyzed endurance exercise paradigm in *daGS > DNase II* overexpressing flies. We observed that upregulation of DNase II improves *Drosophila* endurance when compared with age-matched control flies (Fig. 2i). Importantly, RU486 treatment does not extend *Drosophila* lifespan (Supplementary Fig. 2g). Together, these results show that ubiquitous *DNase II* or neuronal *Sid* overexpression can prolong healthspan.

### Mitophagy and nuclease activity counteract NF-κB-like proinflammatory signaling in aged flies

**Figure 3:**
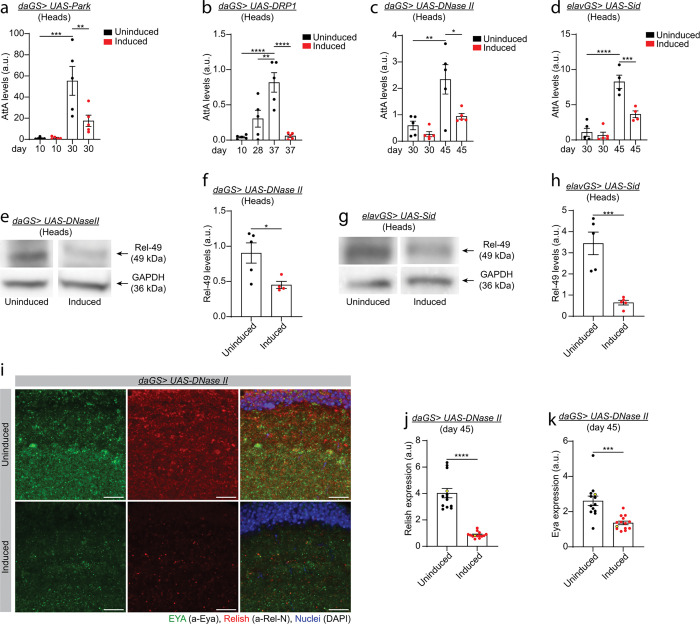
Mitophagy and nuclease activity counteract NF-KB-like proinflammatory signaling in aged flies. **a)** qPCR analysis of *AttA* levels in *daGS> UAS-Park* female flies in heads with or without RU486-mediated transgene induction from day 3 onwards. d10 uninduced n = 5, d10 induced n = 5, d30 uninduced n = 5, and d30 induced n = 5 biological replicates with 10 heads per replicate. d10 uninduced vs d30 uninduced ***p = 0.0002; d30 uninduced vs d30 induced **p = 0.0065. Ordinary one-way ANOVA test with Sidak’s multiple comparisons test. **b)** qPCR analysis of *AttA* levels in *daGS> UAS-DRP1* female flies in heads with or without RU486-mediated transgene induction from day 3 onwards. d10 uninduced n = 5, d28 uninduced n = 5, d37 uninduced n = 5, and d37 induced n = 5 biological replicates with 10 heads per replicate. d28 vs d30 uninduced **p = 0.0045; d10 vs d37 uninduced ****p < 0.0001; d37 uninduced vs d37 induced ****p < 0.0001. Ordinary one-way ANOVA test with Sidak’s multiple comparisons test. **c)** qPCR analysis of *AttA* levels in *daGS> UAS-DNase II* female flies in heads with or without RU486-mediated transgene induction from day 3 onwards. d30 uninduced n = 5, d30 uninduced n = 5, d45 uninduced n = 5, and d45 induced n = 5 biological replicates with 10 heads per replicate. d30 uninduced vs d45 uninduced **p = 0.0024; d45 uninduced vs d45 induced *p = 0.0133. Ordinary one-way ANOVA test with Sidak’s multiple comparisons test. **d)** qPCR analysis of *AttA* levels in *elavGS> UAS-Sid* female flies in heads with or without RU486-mediated transgene induction from day 3 onwards. d30 uninduced n = 5, d30 uninduced n = 5, d45 uninduced n = 4, and d45 induced n = 4 biological replicates with 10 heads per replicate. d30 uninduced vs d45 uninduced ****p < 0.0001; d45 uninduced vs d45 induced ***p = 0.0005. Ordinary one-way ANOVA test with Sidak’s multiple comparisons test. **e)** Western blot detection of Rel-49 levels of day 45 *daGS> UAS-DNase II* female flies in heads with or without RU486-mediated transgene induction. GAPDH was used as a loading control. **f)** Western blot quantification of Rel-49 levels as shown in e. Uninduced n = 5, induced n = 4 biological replicates with 10 heads per sample. d45 uninduced vs d45 induced *p = 0.0318. Unpaired t-test. **g)** Western blot detection of Rel-49 levels of day 45 *elavGS> UAS-Sid* female flies in heads with or without RU486-mediated transgene induction. GAPDH was used as a loading control. **h)** Western blot quantification of Rel-49 levels as shown in g. Uninduced n = 5, induced n = 5 biological replicates with 10 heads per sample. d45 uninduced vs d45 induced ***p = 0.0009. Unpaired t-test. **i)** Representative images (data point in yellow in j) of immunofluorescence staining of *Drosophila* brains (optic lobe) from day 45 *daGS> UAS-DNase II* female flies, showing EYA (green channel, anti-EYA), Relish (red channel, anti-Rel-N), and nuclei (blue channel, DAPI). Scale bar 5mm. **j)** Statistical quantification of Rel expression as shown in i. Uninduced n = 13 and induced n = 14 brains. d45 Unpaired vs d45 induced ****p < 0.0001. Unpaired t-test. **k)** Statistical quantification of Eya expression as shown in i. Uninduced n = 13 and induced n = 14 brains. d45 Unpaired vs d45 induced ***p = 0.0001. Unpaired t-test. RU486 or vehicle was provided in the media at a concentration of 25 mg/ml for all panels except panel a, where the RU486 was 5 mg/ml, in the indicated treatment groups. Data are presented as scatter plots overlaying mean values +/− SEM.

To explore the interplay between age-associated cytosolic mtDNA accumulation and the immune response in aged flies, we set out to examine the impact of *Parkin* and *DRP1* upregulation on immune-related gene expression. Consistent with previous reports^46,47^, aged control flies present higher levels of expression of the antimicrobial peptide (AMP) *AttacinA* (*AttA*) compared to young flies (Fig. 3a and b and Supplementary Fig. 3a and d). Interestingly, whole life *Parkin* overexpression or one-week *Drp1* induction from midlife ameliorates the immune response in heads and thoraxes from old flies (Fig. 3a and b, and Supplementary Fig. 3a and d). To seek further evidence for the role of mitophagy in age-onset immune activation, we analyzed the expression levels of *Turandot A* (*TotA*), another polypeptide gene also activated after bacterial and DNA viral infection^48^. First, we analyzed the expression of *TotA* in aged heads and thoraxes of *daGS > UAS-Parkin* and *daGS > UAS-DRP1* uninduced flies. *TotA* mRNA transcript levels increase from young to aged flies in heads and thoraxes (Supplementary Fig. 3b, c, e, and f). Upon whole life *Parkin* or one-week *DRP1* induction from midlife *TotA* mRNA transcript levels decreased in middle-aged flies (Supplementary Fig. 3b, c, e, and f, respectively). These results demonstrate that stimulating mitophagy, which reduces cytosolic mtDNA, ameliorates the activation of the immune response in old flies.

To test if cytosolic DNA degradation can reduce age-onset immune activation in *Drosophila*, we analyzed the mRNA transcript levels of *AttA* and *TotA* during aging in *DNase II* and *Sid* overexpressing flies and controls. Interestingly, ubiquitous *DNase II* induction reduces the transcript levels of *AttA* and *TotA* in heads and thoraxes of aged flies as compared to age-matched control flies (Fig. 3c and Supplementary Fig. 3g-i). Moreover, neuronal *Sid* upregulation ameliorates the activation of the immune response related genes *AttA* and *TotA* in aged heads as compared to vehicle fed control flies (Fig. 3d and Supplementary Fig. 3j).

Cytosolic DNA is recognized by the conserved *eya* gene via its threonine phosphatase motif^31,32^. EYA recognizes cytosolic DNA and interacts with the NF-κB-like transcription factor Relish that induces the expression of immune-related genes, including antimicrobial peptides (AMPs)^31,49^. Relish is a compound protein with two domains, an N-terminal Rel Homology Domain (RHD), and a C-terminal IkB-like region. Rel activation requires an endoproteolytic cleavage and the translocation of the RHD domain to the nucleus^50^. To further investigate the role of age-associated cytosolic DNA accumulation in immune response activation, we examined by western blotting the levels of the transcription factor Relish (Rel) upon ubiquitous *DNase II* and neuronal *Sid* upregulation. Ubiquitous *DNase II* or neuronal *Sid* upregulation reduces the levels of nuclear Rel (Rel-49) in heads of aged flies (Fig. 3e and g, and quantification in f and h, respectively). Moreover, we analyzed, by IF, Rel levels in brains of *DNase II* overexpressing and control flies. We saw a striking reduction in Rel protein levels in aged fly brains upon whole life *DNase II* upregulation as compared to age-matched control flies (Fig. 3i and quantification in j). Using *daGS > UAS-DNase II* flies, we examined the relationship between DNase II and EYA in aged flies by IF. Remarkably, DNase II upregulation decreases EYA levels in aged brains as compared to control flies (Fig. 3i and quantification in 3k). Overall, our data indicate that age-associated cytosolic mtDNA accumulation triggers the activation of the immune response in aged flies, and that its reduction ameliorates the expression of immune response-related genes.

### Neuronal inhibition of EYA during aging prolongs healthspan

**Figure 4:**
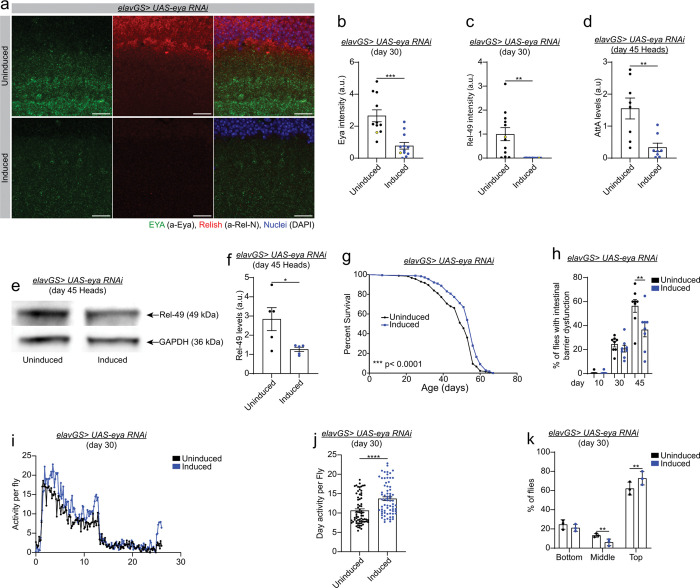
Neuronal inhibition of EYA during aging prolongs healthspan. **a)** Representative images (data point in yellow in b and c) of immunofluorescence staining of *Drosophila* brain (optic lobe) from day 45 *elavGS> UAS-eya RNAi* female flies with RU486-mediated transgene expression from day 3 onwards, showing Eya (green channel, anti-Eya), Relish (red channel, anti-Rel-N), and nuclei (blue channel, DAPI). Scale bar 10 mm. **b)** Quantification of EYA intensity levels as shown in a. Uninduced n = 11 and induced n =12 brains. ***p = 0.0002. Unpaired t-test. **c)** Quantification of Rel-49 intensity levels as shown in a. Uninduced n = 12 and induced n =11 brains. **p = 0.0022. Unpaired t-test. **d)** qPCR analysis of *AttA* levels in *elavGS> UAS-eya RNAi* female flies in heads with or without RU486-mediated transgene induction from day 3 onwards. Uninduced n = 8 and induced n = 8 biological replicates with 10 heads per replicate. **p = 0.0030. Unpaired t-test. **e)** Western blot detection of Rel-49 levels of day 45 *elavGS> UAS-eya RNAi* female flies in heads with or without RU486-mediated transgene induction. GAPDH was used as a loading control. **f)** Western blot quantification of Rel-49 levels as shown in e. Uninduced n = 5, induced n = 5 biological replicates with 10 heads per sample. *p = 0.0320. Unpaired t-test. **g)** Survival curves of *elavGS> UAS-eya RNAi* females with or without RU486-mediated transgene induction from day 3 onward. See Table S4 for statistical analysis. **h)** Percentage of flies with Intestinal barrier integrity loss during aging (Smurf assay) of *elavGS> UAS-eya RNAi* females with or without RU486-mediated transgene induction since day 3 onward. n = 300 flies on day 10. d10 uninduced vs d30 uninduced ***p = 0.0001; d10 uninduced vs d45 uninduced ****p < 0.0001; d30 uninduced vs d45 uninduced ****p < 0.0001; d45 uninduced vs d45 induced ** p = 0.0019. Ordinary one-way ANOVA test with Sidak’s multiple comparisons test. **i)** Spontaneous physical activity of 30-day-old *elavGS> UAS-eya RNAi* female flies with or without RU486-mediated transgene induction from day 3 to the day of the assay. **j)** Quantification of daytime physical activity of *elavGS> UAS-eya RNAi* female flies with or without RU486 from day 3 to the day of the assay. Uninduced n = 3 and Induced n = 3 vials with 10 flies per vial. ****p < 0.0001. Unpaired t-test. **k)** Climbing index as a measure of endurance of 30-day-old *elavGS> UAS-eya RNAi* female flies with or without RU486-mediated transgene induction from day 3 to day 30. Bottom represents the lower 1/3 of the cylinder, middle represents the middle 2/3 of the cylinder, and top represents the upper 1/3 of the cylinder. Uninduced n = 3 and induced n = 3 vials with 3 vials with at least 60 flies per vial. middle uninduced vs induced **p = 0.0083; top uninduced vs induced **p = 0.0018. 2-way ANOVA test with Sidak’s multiple comparisons test. RU486 or vehicle was provided in the media at a concentration of 50 mg/ml in the indicated treatment groups. Data are presented as scatter plots overlaying mean values +/− SEM.

To better understand the potential role of EYA in aging, we analyzed *eya* transcript levels in young (day 10), middle-aged (day 28 and 35), and old (day 42) wild type flies. *eya* mRNA levels increase more than 4-fold from young to middle-aged and old flies and decrease by 37 percent after day 35 in wild type heads (Supplementary Fig. 4a). We also analyzed the *eya* mRNA levels in thoraxes and showed that *eya* transcript levels slightly increase from day 10 to day 35 and 42 (Supplementary Fig. 4b). Since, *eya* transcript levels increase dramatically in heads of aged flies, we examined whether *eya* neuronal knockdown could ameliorate the activation of the immune response in aged fly heads. First, we validated that RNAi against *eya* inhibits the expression of EYA protein in aged flies. Confocal analysis shows that EYA protein levels decrease in *elavGS > UAS-eya-RNAi* induced flies as compared to uninduced control flies (Fig. 4a and quantification in b). Next, we found that neuronal downregulation of *eya* decreases *AttA* mRNA transcript levels in aged fly heads (day 45) compared to control flies and shows a trend towards a reduction in *TotA* mRNA transcript levels (Fig. 4d and Supplementary Fig. 4c). To seek evidence that EYA regulates the immune response through its interaction with Rel, we set out to examine the protein levels of Rel in heads of *eya* neuronal knockdown flies. Interestingly, neuronal inhibition of *eya* reduces Rel protein levels in brains of aged flies (Fig. 4a and quantification in c, and 4e and quantification in f). Importantly, neuronal *eya* knockdown does not change cytosolic mtDNA levels in old flies (Supplementary Fig. 4d). Together, these results demonstrate that EYA contributes to age-onset neuroinflammation.

To deepen our understanding of the role of EYA in aging, we set out to analyze the healthspan of *eya* neuronal knockdown flies. In the first place, we examined the lifespan of *elavGS > UAS-eya-RNAi* flies and observed that neuronal *eya* knockdown prolongs *Drosophila* lifespan in several trials (Fig. 4g and Supplementary Table s3). Importantly, *eya* neuronal downregulation improves intestinal barrier function (Fig. 4h), spontaneous activity (Fig. 4i and j), and endurance exercise capacity (Fig. 4k). Collectively, these data reveal that neuronal *eya* downregulation extends *Drosophila* lifespan, delays age-onset intestinal pathology and improves healthspan.

### Neuronal inhibition of EYA slows hallmarks of brain aging

**Figure 5:**
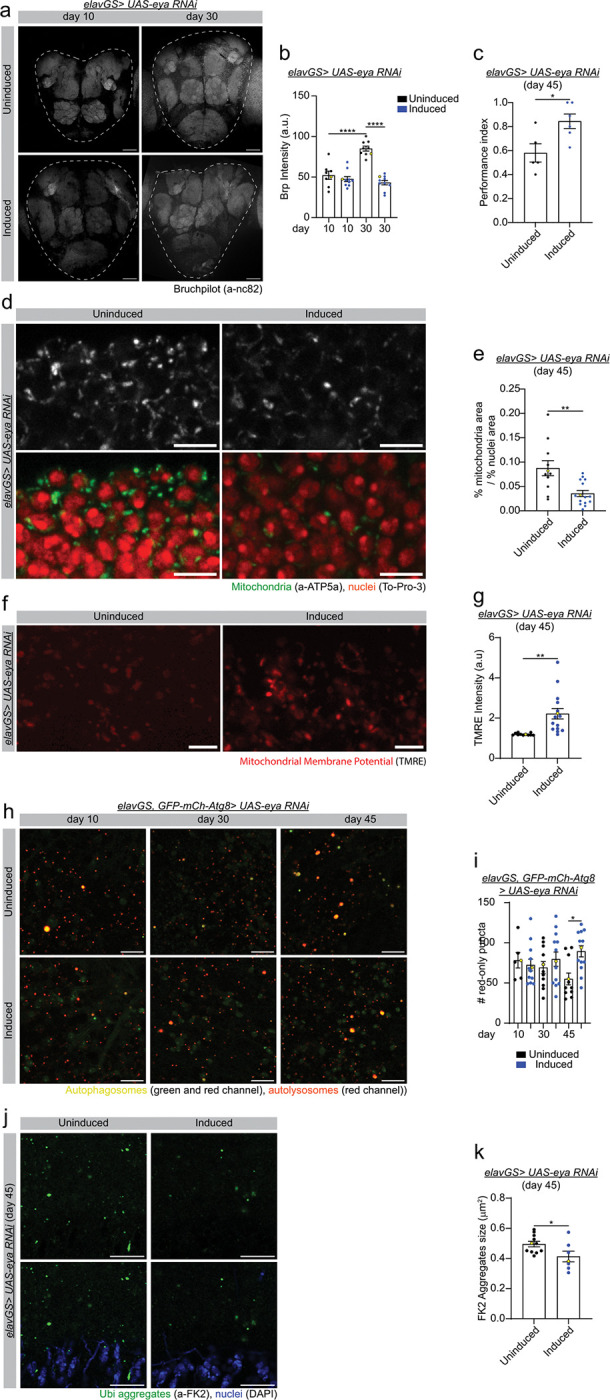
Neuronal inhibition of EYA slows hallmarks of brain aging. **a)** Representative images (data point in yellow in b) of immunofluorescence staining of *Drosophila* brain from day 10 and 30 *elavGS> UAS-eya RNAi* female flies with or without RU486-mediated transgene expression from day 3 onwards, showing Bruchpilot (gray channel, anti-nc82). Scale bar 50 mm. **b)** Quantification of Brp expression as shown in a. d10 Uninduced n = 8, d10 induced n = 10, d30 uninduced n = 9, and d30 induced n = 10 brains. d10 uninduced vs d30 uninduced ****p < 0.0001; d30 uninduced vs d30 induced ****p < 0.0001. Ordinary one-way ANOVA test with Sidak’s multiple comparisons test. **c)** Performance index in olfactory aversion training in 45-day-old *elavGS> UAS-eya RNAi* female flies with or without RU486, assessed by the number of flies avoiding a shock-associated odor versus the total number of flies participating in the assay. Uninduced n = 5 and induced n = 6 vials with more than 10 flies per vial. Uninduced vs induced *p = 0.0225. Unpaired t-test. **d)** Representative images (data point in yellow in e) of immunofluorescence staining of *Drosophila* brain from day 45 *elavGS> UAS-eya RNAi* female flies with or without RU-486-mediated transgene expression from day 3 onwards, showing mitochondria (green channel, a-ATP5a), and nuclei (red channel, To-Pro-3). Scale bar 5 mm. **e)** Quantification of ATP5a amount as shown in a. Uninduced n = 11 and induced n = 15 brains. Uninduced vs induced **p = 0.0021. Unpaired t-test. **f)** Representative images (data point in yellow in g) of immunofluorescence staining of *Drosophila* brain from day 45 *elavGS> UAS-eya RNAi* female flies with or without RU486-mediated transgene expression from day 3 onwards, showing mitochondrial membrane potential (red channel, TMRE). Scale bar 5 mm. **g)** Quantification of TMRE expression as shown in c. Uninduced n = 11 and induced n =15 brains. Uninduced vs induced **p = 0.0028. Unpaired t-test. **h)** Representative images (data point in yellow in i) of immunofluorescence staining of *Drosophila* brains from young (d10), middle (30), and old (d45) age *elavGS, GFP-mCherry-Atg8a> UAS-eya-RNAi* female flies with or without RU486-mediated transgene expression from day 3 onwards, showing autophagosomes (green and red channel), and autolysosomes (red channel). Scale bar 10 mm. **i)** Quantification of autolysosome number as shown in e. d10 uninduced n = 6, d10 induced n = 11, d30 uninduced n = 11, d30 induced n =14, d45 uninduced n = 11, d45 n= 13 brains. d45 uninduced vs d45 induced *p = 0.0134. Ordinary one-way ANOVA test with Sidak’s multiple comparisons test. **j)** Representative images (data point in yellow in j) of immunofluorescence staining of *Drosophila* brain from day 45 *elavGS> UAS-eya RNAi* female flies with or without RU486-mediated transgene expression from day 3 onwards, showing ubiquitin aggregates (green channel, anti-FK2) and nuclei (blue channel, DAPI). Scale bar 10 mm. **k)** Quantification of FK2 aggregate size as shown in g. Uninduced n = 11 and induced n = 7 brains. Uninduced vs induced *p = 0.0366. Unpaired t-test. RU486 or vehicle was provided in the media at a concentration of 50 mg/ml in the indicated treatment groups. Data are presented as scatter plots overlaying mean values +/− SEM.

Age-related memory impairment (AMI) is associated with alterations in neuronal physiology; more specifically in synaptic connectivity^51,52^. Studies in *Drosophila* have shown that an increase in the size of the pre-synaptic active zone is associated with a decline in memory and sleep disruption^53,54^. *Drosophila*, as well as other insects, shares high levels of homology in the design and function of the olfactory nervous system with mammals. Bruchpilot (BRP) shows homology to the active zone human protein ELKS/CAST/ERC^55^. It has been demonstrated that aging increases the active zone structure and the expression of the active zone protein BRP^53^. Here, to better characterize the effect of *eya* knockdown on synaptic aging, we analyzed the levels of BRP in *eya* knockdown flies and controls. Importantly, as previously reported^53^, we saw an increase in BRP protein levels in control middle-aged (day 30) flies when compared with young control flies (Fig. 5a and quantification in b, and Supplementary Fig. 5a). Interestingly, aged brains with reduced levels of EYA showed a reduction in BRP at day 30 when compared with age-matched control flies (Fig. 5a and quantification in b, and Supplementary Fig. 5a). Next, we examined whether neuronal EYA activity contributes to cognitive decline during aging. Remarkably, neuronal inhibition of EYA improves performance in the olfactory aversion training assay (Fig. 5c). These results indicate that neuronal *eya* knockdown suppresses age-related memory impairment and delays synaptic aging.

In recent years, significant attention has been focused on the cellular hallmarks of aging^1^, including hallmarks of brain aging^56^. To provide a mechanistic understanding of how neuronal inhibition of EYA slows brain aging, we examined several key cellular hallmarks of aging. In the first place, we examined the impact of neuronal *eya* knockdown on markers of mitochondrial homeostasis. We have previously shown that dysfunctional mitochondria accumulate in aged fly brain tissue^57,58^. Importantly, we find that neuronal *eya* downregulation decreases mitochondrial content in aged brains as compared to controls (Fig. 5d and quantification in e). Next, we set out to examine the impact of neuronal *eya* inhibition on mitochondrial activity during aging, using the mitochondrial membrane potential potentiometric dye TMRE (tetramethylrhodamine, ethyl ester). We observed that *eya* downregulation in neurons significantly improves mitochondrial membrane potential in aged brains (Fig. 5f and quantification in g). Together, these data show that neuronal EYA activity compromises mitochondrial homeostasis during brain aging.

Disabled autophagy and loss of protein homeostasis (proteostasis) are thought to be primary hallmarks of aging, which unambiguously drive the aging process^1^. Hence, we next sought to determine whether neuronal *eya* downregulation could improve autophagy and/or proteostasis in aged *Drosophila* brains. To evaluate autophagic activity in the aging brain, we used a reporter line expressing GFP-mCherry-Atg8a (“Atg8a-tandem”) ubiquitously under the control of the endogenous Atg8a promoter^59^. As autophagosomes fuse with lysosomes, GFP signal on the Atg8a tandem protein is quenched due to its sensitivity to low pH. Remaining mCherry-only foci indicate autolysosomal activity. Using this approach, we find that neuronal *eya* inhibition results in a significant increase in autolysosomes in aged brains (day 45) compared to control flies (Fig. 5h and quantification in i as shown in Supplementary Fig. 5b). To examine the impact of *eya* knockdown on protein homeostasis, we analyzed the accumulation of protein aggregates in aged brains of neuronal *eya* knockdown flies and controls. IF microscopy analysis shows that brains with reduced levels of EYA present smaller ubiquitin-containing protein aggregates (Fig. 5j and quantification in k, and Supplementary Fig. 5c and quantification in d). Together, our results indicate that neuronal *eya* inhibition improves proteostasis and autophagy in aged *Drosophila* brains, two of the major primary hallmarks of aging.

## Discussion

Studies in both vertebrate and invertebrate models have shown that mitophagy can counteract aging and prolong lifespan^38–40,58,60,61^. These observations strongly support a model in which dysfunctional mitochondria, within aged cells, drive pathology and limit lifespan. Yet, a clear understanding of the mechanisms that underlie age-onset health decline upon mitochondrial dysfunction is lacking. Recent work has shown that mitophagy can dampen age-onset cGAS/STING-driven neuroinflammation in mice^22^. Moreover, treatment with STING inhibitors can reduce age-associated neuroinflammation and improve cognition^27^. Together, these findings support a model in which cytosolic DNA, originating from dysfunctional mitochondria, accumulates in certain aged brain cells, driving cGAS/STING mediated-neuroinflammation and disrupting neurological function. Here, we have extended these findings to show that cytosolic mtDNA accumulates broadly in fly brains, muscle tissue and whole bodies. Moreover, we provide direct evidence that cytosolic DNA and associated pro-inflammatory signaling limits organismal lifespan and healthspan. We show that genetic induction of nuclease activities can dampen age-onset inflammation and prolong organismal healthspan. Critically, we show that inducing DNase II or Sid reduces cytosolic mtDNA levels in aged flies. It should be noted, however, that we cannot exclude the possibility that the nuclease-mediated degradation of additional nucleic acids could contribute to observed phenotypes.

The finding that the cGAS/STING signaling pathway is a critical driver of neurodegenerative processes during aging^62^ raises the question of whether additional interventions targeting the immune sensing of DNA can counteract aging-related pathophysiology. In addition to the Toll receptor and the Toll signaling pathway, the *Drosophila* immune response is regulated by another evolutionarily conserved signaling cascade, the immune deficiency (Imd) pathway, which activates Relish/NF-κB^63^. The fly EYA protein acts in a cascade that senses undigested cytosolic DNA and activates the immune response by binding to the I-kappa-B kinase beta (IKKβ), component of the IKK phosphorylation complex that phosphorylates Relish, and Relish^31^. We show that neuronal inhibition of EYA dampens age-onset Relish/NF-κB activity, slows several markers of brain aging and prolongs organismal lifespan. As mammalian EYA4 enhances the innate immune responses against DNA by activating NF-κB^32^, it stands to reason that EYA4 represents an attractive target to slow brain aging and prolong healthspan in mammals. A challenge to consider, in this regard, is that while interventions that reduce the immune sensing of DNA may promote healthspan in laboratory animals, there could be detrimental consequences outside of a laboratory setting.

One of the major findings from our study is that inhibiting EYA in aging neurons ameliorates a number of hallmarks of brain aging, including disabled autophagy which has been designated as a primary hallmark of aging^1,56^. Our data doesn’t refute the idea that disabled autophagy precedes cytosolic mtDNA accumulation and associated pro-inflammatory signaling during aging. Rather, the simplest interpretation of our findings is that neuronal EYA activity, in response to cytosolic DNA, exacerbates autophagy impairments in aged cells. In turn, disabled autophagy has been shown to contribute to synaptic aging and age-onset cognitive decline^51,53^. Hence, a plausible interpretation of our findings is that EYA activity in aging neurons drives cognitive decline via disabled autophagy. We show that neuronal inhibition of EYA counteracts NF-κB-like proinflammatory signaling in aged brains. Previous studies have shown that, under certain conditions, NF-*κ*B signaling activates the expression of autophagy inhibitors and represses activators of autophagy^64^. Hence, our working hypothesis is that reduced NF-κB-like proinflammatory signaling in aged brains, upon neuronal EYA inhibition, leads to improved brain autophagy. Antimicrobial peptides (AMPs) often target the cellular membrane or cell wall of gram-positive and gram-negative bacteria, viruses, and fungi and have different mechanisms of action including membrane permeabilization^65^. Mitochondrial and bacterial membranes share some similarities like lipid composition^66^. These similarities could make the mitochondrial membrane a suitable target for AMPs. Recent studies have demonstrated that AMPs change mitochondrial membrane permeability and induce apoptosis^67^. It is possible, therefore, that reduced AMP levels in response to EYA inhibition may lead to improved mitochondrial homeostasis in aged brains. Future work could focus on finding ways to dampen NF-κB-like proinflammatory signaling in aged brains without compromising pathogen susceptibility.

## Materials & Methods

### Fly Stocks

The fly strain *Elav–GeneSwitch* (*elavGS*) was provided by H. Keshishian (Yale University, New Haven, CT, USA). *daughterless-GeneSwitch* (*daGS*) was provided by H. Tricoire (Université Paris Diderot–Paris7, Paris, France). *UAS-Parkin-HA* (*UAS-Park*) was provided by L. Pallanck (University of Washington, Seattle, WA, USA). *UAS-DRP1* was provided by J. Chung (Korea Advanced Institute of Science and Technology, Republic of Korea). GFP-mCherry-Atg8a was provided by Eric Baehrecke (University of Massachusetts Medical School, Worcester, MA, USA). *UAS-eya RNAi* (28733), *sqh-mito-EYFP* (7194), *UAS-mito-HA-GFP* (8442), *UAS-mCD8::GFP* (32185), and *w*^*1118*^ (3605) were acquired from the Bloomington *Drosophila* Stock Center.

### Fly Husbandry and Lifespan Analysis

Flies were reared in vials containing cornmeal medium (1% agar, 3% yeast, 1.9% sucrose, 3.8% dextrose, 9.1% cornmeal, 1.1% acid mix (41.8% Propionic acid plus 4.15% Phosphoric acid in vol/vol), and 1.5% methylparaben (10% methylparaben in ethanol), all concentrations given in wt/vol). Flies were collected under light nitrogen-induced anesthesia and housed at a density of 30 female flies per vial. All flies were kept in a humidified, temperature-controlled incubator with a 12 h on/off light cycle at 25°C. RU486 was dissolved in ethanol and administered in the media while preparing food. RU486 concentration is given in mg/mL in the figure legend for each treatment. Flies were flipped to fresh food containing vial every 2–3 days and scored for death.

### Transgenic flies generation

*UAS-Sid* (UAS-CG9989) and *UAS-DNase II* (UAS-CG7780) fly lines were generated by phiC31 integrase mediated transformations of flies harboring an intergenic attP site in chromosome 2R (“attP33”)^68^ with pUASTattB plasmids^69^ edited with corresponding cDNA sequences. Sid cDNA was amplified from LP02841 (Drosophila Genomics Resource Center Stock 12973), DNAseII cDNA was amplified from GH10876 (DGRC Stock 6248) using standard PCR protocols. All plasmids were validated by sequencing prior to transformation. Cloning primers (utilizing a SfiI recognition sequence: sid_F-CGCAGGGCCGGACGGGCCAGATGCCCGATCTGAAGTATATG, sid_R-CGCAGGGCCCCAGTGGCCCAAATACCACTTTATATTTATTTTAATATGC, DNase II_F-CGCAGGGCCGGACGGGCCCAACTTGAAGGTTGTACAATGCG, DNase II_R-CGCAGGGCCCCAGTGGCCCAATTTTCTTATGCATTAAATGTAATGC.

### Spontaneous Physical Activity Assay

10 adult female flies were placed in a *Drosophila* activity monitor (TriKinetics). Movements were recorded continuously every 30s under normal culturing conditions for 36 h on a 12 h:12 h dark:light cycle. Bar graphs represent mean activity per fly per hour, and the scatterplot shows spontaneous activity per fly during a 12 h:12 h dark:light cycle. Triplicate samples were used for each activity measurement.

### Climbing Activity Assay

At least 60 adult female flies were placed in a 100 mL glass cylinder. Cylinders were tapped quickly, and flies were allowed to settle for 1 min. This step was repeated 8 times. Then the cylinder was tapped quickly and after 1 min, the number of flies in the upper, middle, and lower 1/3rd parts of the cylinder was recorded.

### Intestinal Barrier Dysfunction Assay (Smurf Assay)

Intestinal barrier dysfunction was performed as previously described in^44^ Rera et al. (2012). Flies were aged in normal or RU486 containing food until the day of the assay. The day before the assay, flies were transferred to new vials containing standard medium with 2.5% wt/vol F&D blue dye # 1 (SPS Alfachem) and Ethanol for control flies or RU486 for experimental flies. Flies were kept in this medium for at least 16 hours. Flies with dye coloration outside the gut were counted as flies with loss of gut integrity (Smurf fly).

### Olfactory Training

Aversion training was performed with modifications as described in^45^ Malik et al. (2014) using a system from MazeEngineers (Conduct Science). Briefly, flies were exposed under low red-light conditions to a neutral odor (3-octanol OCT) by air pump in a training chamber for one minute in a series of twelve 65-V and 0.2 mA electrical-shocks for 1.25s followed by 3.75s of rest. Flies recovered for one hour before being placed in a T-maze with trained odor on one side and a second neutral odor (4-methylcyclohexanol, MCH) on the other side of the maze. After two minutes of exploration under red-light conditions, flies in either chamber of the maze were scored. Performance index was calculated by dividing the number of flies avoiding OCT by the number of participants (OCT + MCH flies).

### Cellular Fractionation and cytosolic and nuclear DNA isolation

Cellular fractionation was performed as described in^70^ Mosley and Baker (2022) with modifications. Heads (25 heads) or thoraxes (20 thoraxes) were gently homogenized in 200 μL of cold Mitochondrial Isolation Medium (250 mM sucrose, 10 mM TrisHCl (pH 7.4), 0.15 mM MgCl2). Samples were spun for 5 seconds to remove fly debris and then spun at 15000Xg for 15 min. at 4°C for mitochondria and nuclei purification (nuclear and mitochondrial fraction). Supernatant (cytosolic fraction) was collected (170 mL) in a new 1.5 μL vial and pellet was resuspended in 100 μL Nuclear Isolation Medium (10 mM HEPES-KOH pH 7.5; 2.5 mM MgCl_2_; 10 mM KCl). Nuclei and cytosolic fraction were incubated with 1 μL of Proteinase K (10 mg/mL) (Fisher-Scientific, cat# BPI1700–100) for 1 hour at 55°C. Proteinase K was inactivated by incubating the samples for 10 min. at 95°C. One volume of phenol:chloroform:isoamyl alcohol (25:24:1) was added to each sample and shaken by hand thoroughly for approximately 20 seconds. Then, samples were spun at 16000Xg for 15 min at room temperature. The upper phase (aqueous phase) containing DNA was transferred to a new 1.5 mL vial, and 1 μL of glycogen, 0.5 volumes of NH_4_OAc, and 2.5 volumes (samples + NH_4_OAc) of EtOH were added. Samples were kept at −20°C overnight. The following day, samples were centrifuged at 15000Xg for 30 min. at 4°C. Pellet (DNA) was washed twice with 70% EtOH and resuspended in 20 μL of TE Buffer (1 mM EDTA & 10 mM PH 8 Tris-Cl). Cytosolic fraction was tested for mitochondria contamination by taking 10 μL each of the cytosolic and nuclear/mitochondria fraction and analyzing the samples by western blot against the mitochondrial protein VDAC1 and the cytosolic protein Actin (Supplementary Fig. 1A).

### RNA Extraction, cDNA Synthesis and quantitative PCR (qPCR)

10 heads or 5 thoraxes were homogenized in 100 μL of ice cold Trizol (Thermo Fisher Scientific cat# 15596018) for RNA extraction. Samples were incubated at room temperature for 10 min. Then, 20 μL of Chloroform (Millipore-Sigma cat# C2432–500ML) was added to the samples and shaken vigorously by hand for 20 seconds. Samples were incubated for another 10 min. at room temperature, then centrifuged at 12000Xg for 15 min. at 4°C. 45 μL of the upper phase, containing the RNA, was transferred to a new 1.5 mL vial. For head samples, 4.5 μL of 3.5 M Sodium Acetate (Fisher Scientific cat# R1181) and 2 μL of 20 mg/mL RNA Grade Glycogen (Fisher Scientific cat # R0551) were added to the samples. Then, 50 μL of Isopropyl Alcohol (Fisher Scientific cat# 02–003-133) was added to the samples and briefly vortexed to mix. Samples were spun at 12000Xg for 10 min. at 4°C. Pellet (RNA) was washed with 200 μL of 75% EtOH, air dried for 5 min, resuspended in 20 μL ddH20, and incubated at 55°C for 10 min.

cDNA synthesis was carried out using the First Strand cDNA Synthesis Kit from Thermo Fisher Scientific (cat# K1621, K1622). PCR was performed with PowerUP SYBR Green Master Mix (Ref#A25777, Applied Biosystems) on a BioRad Real Time PCR system. Cycling conditions were as follows: 95°C for 10 minutes; 95°C for 15 s then 60°C for 60 s, cycled 40 times, and equalized amplicons of Actin5C were used as a reference to normalize for cytosolic mitochondrial DNA, and GAPDH was used as a reference for gene expression analysis. Primers sequences used were as follows:

GAPDH_F: CTCCACCACAACTCGGCTC and GAPD_R: TAAATTCGACTCGACTCACGGT

Act5C_F: TTGTCTGGGCAAGAGGATCAG and Act5C_R: ACCACTCGCACTTGCACTTTC

### COI

_F: GAATTAGGACATCCTGGAGC and COI_R: GCACTAATCAATTTCCAAATCC

ND2_F: AAAAAGTGGAGCCGCTCC and ND2_R: GTTTGATTTAATCCTCCAATAGCTCC

DNase II_ F: AGGATGAAGCTGGAAACGATG and DNase II_R: CAGGTGTCATAGTTCTGGCTG

Sid_F: TTCCATCTACAAGGCTTATCGC and Sid_R: TTGTGTTGCTCTTCCCTCG

Eya_F: GTCAGCTCGGACGACAAT and Eya_R: GTGCCAACATTTCCACGATAG

AttA_F: CTCCTGCTGGAAAACATC and Atta_R: GCTCGTTTGGATCTGACC

TotA_F: CCCAGTTTGACCCCTGAG and TotA_R: GCCCTTCACACCTGGAGA

### Western Blot

Heads (10 heads per sample) were homogenized in 100 μL of Lysis Buffer (PBS 1X, Protease Inhibitors 1X, NuPAGE LDS Sample Buffer 1X, and DTT (Dithiothreitol) 0.05M). Samples were incubated for 5 min. at 95°C and centrifuged at 16000Xg for 5 min. at 4°C. 10 μL of samples were separated by SDS-PAGE gels, and proteins were transferred to Nitrocellulose membranes. Membranes were probed with antisera against: rabbit anti-GAPDH (Novus Biologicals cat# NB100–56875), mouse anti-actin peroxidase conjugated 1:15000 (Sigma cat# A3854), mouse anti-VDAC1/Porin 1:10000 (ab14734, Abcam), mouse anti-Relish-N 1:1000 (DSHB cat# 21F3). Anti-Rabbit or anti-Mouse Horseradish peroxidase conjugated antibodies were used for detection at a 1:10000 dilution. Amersham ECL Prime Western Blotting Detection Reagent (GE Life Sciences) was used to visualize the presence of horseradish peroxidase, and the chemiluminescent signal was recorded using Syngene Pxi Western Blot Imager. Image analysis was done using ImageJ.

### Muscle and Brain Immunostaining

For muscle staining, flies were fixed in 3.7% formaldehyde in PBS for 20 minutes. After fixation, hemithoraxes were dissected and fixed again for 5 min. Brains were dissected directly in cold PBS and fixed in 3.7% formaldehyde in PBS for 20 min. at RT. For Brp staining, brains were dissected in saline medium (NaCl 103 mM, KCl 3 mM, TES 5 mM, trehalose 10 mM, glucose 10 mM, sucrose 7 mM, NaHCO_3_ 26 mM, NaH_2_PO_4_ 1 mM, CaCl_2_ 1.5 mM, MgCl_2_ 4 mM adjusted to 280 mOsm) and fixed in 3.7% formaldehyde in PBS for 20 min. at RT. Brains and hemithoraxes were then rinsed 3 times for 10 min. with 0.2% Triton X-100 in PBS (PBST) and blocked in 3% BSA in PBST (PBST-BSA) for 1 hour. Primary antibodies were diluted in PBST-BSA and incubated overnight at 4°C, except for Brp, which was incubated for more than 48 hours. Primary antibodies used were: mouse-anti-FK2 1:250 (04–263, Millipore Sigma); mouse-anti-ATP5A1 1:250 (ab14748, abcam); anti-EYA 1:10 (10H6, DSHB); anti-BRP 1:100 (nc82, DSHB); and anti-Relish-N 1:50 (RB 14–0024-20, RayBiotech). Hemithoraxes and brains were then rinsed 3 times in PBST for 10 min. and incubated with the secondary antibodies and/or stains at room temperature for 3 hours. Secondary antibodies used were: anti-rabbit or anti-mouse AlexaFluor-488 1:500 (Invitrogen); anti-rabbit or anti-mouse AlexaFluor-568 1:500 (Invitrogen); To-Pro-3 (1:1000, Invitrogen) or DAPI (300nM, Invitrogen) for DNA staining. Finally, samples were rinsed 3 times with PBST for 10 min. and mounted in Vectashield Mounting Medium (Vector Lab). Images were acquired using a Zeiss LSM 880 Airyscan Confocal Microscope.

### Muscle and Brain Low Permeability Immunostaining

Low permeability immunostaining was performed as described in^33^ Sato et al. (2021) with modifications. For muscle staining, flies were fixed in fixation solution (3.7% formaldehyde in PBS) for 20 min. After fixation, hemithoraxes were dissected and fixed for another 2.5 hours in cold-ice fixation solution. For brain staining, flies were dissected directly in cold PBS and fixed for 2.5 hours in ice-cold fixation solution. After fixation, samples were washed 5 times in cold PBS on ice for 10 mins each. Brains and hemithoraxes were dehydrated using the following EtOH series solutions: 5%, 10%, 20%, 50%, 70%, and 100% for 5 min. each on ice. Samples were then washed once more with 100% EtOH for 10 min on ice and incubated in new 100% EtOH at −20°C overnight. The next day, brains and hemithoraxes were rehydrated in ice-cold EtOH solutions (70%, 50%, 20%, 10%, and 5%) for 5 min each. Samples were then washed four times with ice-cold PBS and three times with room temperature PBS. Brains and hemithoraxes were incubated in permeabilization buffer (Tween 20 0.1% V/V plus Triton X-100 0.01% V/V in PBS) for 5 min. Samples were rinsed with PBS and washed 3 times for 10 min. each in PBS. Next, samples were incubated in blocking buffer (10% normal goat serum in PBS) for 1 hour. Primary antibodies used were: mouse-anti-dsDNA 1:500 (ab27156, Abcam) and chicken anti-GFP 1:1000 (GFP1010, Aves Labs). Primary antibodies were diluted in PBS and incubated for 3 days at 4°C. Hemithoraxes and brains were then washed 3 times in PBS and 3 times in washing buffer (Tween 20 0.05% V/V in PBS) for 10 min each. Samples were incubated with the appropriate secondary antibody for 3 days. Finally, samples were washed 3 times in washing buffer for 10 min. and mounted in Vectashield Mounting Medium (Vector Lab). Images were acquired using a Zeiss LSM 880 Airyscan Confocal Microscope.

### TMRE Staining

Flies were anesthetized and dissected in cold Drosophila Schneider’s Medium (DSM). Brains were incubated in TMRE staining solution (100 nM TMRE (Thermo Fisher Scientific cat# T669) in DSM) for 12 min. at room temperature. After staining samples were rinsed once in wash solution (25 nM TMRE in DSM) for 30 s. Brains were mounted in wash solution. Images were acquired using a Zeiss LSM 880 Airyscan Confocal Microscope with the same settings for laser intensity and gain.

### Image Analysis

Images in Fig. 1a, b, h, and k, and Fig. 2a and j were acquired using a Zeiss LSM 880 confocal microscope with Airyscan. For muscles, images were cropped to 21.25 X 13.12 (WXL) microns. For brains, images were cropped to 10.78 X 6.65 (WXL) microns. dsDNA counts were analyzed by quantifying the number of dots outside of mitochondrial staining (GFP).

Images in Supplementary Fig. 1a were acquired with a Zeiss LSM 880 confocal microscope with Airyscan microscopy. Images were cropped to 21.25 X 13.12 (WXL) microns for further analysis. Mitochondria morphology and dsDNA were segmented using the ImageJ Plugin Trainable Weka Segmentation. Segmented images were analyzed for dsDNA colocalization using the ImageJ plugin JACoP (Just Another Colocalization Plugin). Manders’ Colocalization Coefficient was applied to quantify the proportion of dsDNA that colocalizes with mitochondria.

Images in Fig. 3i, 4b, 5h, and j, and Supplementary Fig. 5b and c were acquired with a Zeiss LSM 880 confocal microscope and cropped to 55.35 X 55.35 (WXL) microns. EYA and Rel intensity analysis were quantified in ImageJ using the same threshold limits, and the mean gray value was quantified for each image and normalized to nuclei (To-Pro-3) intensity. FK2 aggregate size and number were quantified in the image using the “analyze particle” tool. Particles smaller than 0.05 μm^2^ were discarded.

Images in Fig. 5d were acquired with a Zeiss LSM 880 confocal microscope and cropped to 23.81X14.76 (WXL). ATP5a percentage was analyzed in ImageJ and normalized to the nuclei percentage.

Images in Fig. 5a were acquired with a Zeiss LSM 880 confocal, using identical settings conditions for each image, and cropped to 428.05X446.96 (WXL) microns. Brp intensity was quantified in the central brain by calculating the mean gray value in ImageJ for each image.

For TMRE staining (Fig. 5f), images were acquired in a Zeiss LSM 880 confocal microscope using identical settings for each condition. Images were cropped to 35.71 X 22.14 (WXL) microns. TMRE intensity was quantified using ImageJ software.

Autolysosomes in Fig. 5h/Sup Fig. 5b were quantified using the mitoQC counter plugin on ImageJ^71^. Briefly, Red dots (autolysosomes) are quantified based on the difference in the intensity profile between the red and green channels.

### Statistical Analysis

GraphPad Prism 10 was used to perform the statistical analysis and graphical display of the data. Statistical significance is expressed as p-values as determined by two-tailed tests. A Gaussian distribution with parametric distribution was used when samples reached the distribution criteria, or non-parametric distribution was used when samples did not reach the criteria for Gaussian distribution. For comparisons between two groups, an unpaired *t*-test was used. For comparisons of more than two groups, one-way ANOVA with Šídák correction or Tukey and Dunnett test was performed. Kruskal-Wallis tests with Dunn’s multiple comparisons post hoc tests were used when data do not meet the criteria for one-Way ANOVA analysis. When performing grouped analyses with multiple comparisons, two-way ANOVAs with Šídák’s multiple comparisons test were performed. Scatter plots with bars depict mean ± SEM. p values are annotated in each figure legend. The number (n) of biological samples used in each experiment and what n represents can be found in each figure legend.

Log-rank (Mantel-Cox) test was used for survival curves comparison. Average median survival is the time point at which the probability of survival equals 50%. Detailed statistical analysis and the difference between survival curves can be found in the supplementary tables.

## Supplementary Material

Supplementary Files

This is a list of supplementary files associated with this preprint. Click to download.

• SupFig3.pdf

• SupFig2.pdf

• Tables4.pdf

• SupFig1.pdf

• TableS3.pdf

• Tables2.pdf

• SupFig4.pdf

• SupFig5.pdf

• Tables1.pdf
